# Fundamentals of end-of-life communication as part of advance care planning from the perspective of nursing staff, older people, and family caregivers: a scoping review

**DOI:** 10.1186/s12912-023-01523-2

**Published:** 2023-10-06

**Authors:** Fran B.A.L. Peerboom, Jolanda H.H.M. Friesen-Storms, Bénédicte J.E.G. Coenegracht, Sabine Pieters, Jenny T. van der Steen, Daisy J.A. Janssen, Judith M.M. Meijers

**Affiliations:** 1 Zuyderland Medical Center, Dr. H. van der Hoffplein 1, Sittard-Geleen, 6162 BG The Netherlands; 2 Department of Health Services Research, Care and Public Health Research Institute, Maastricht University, Duboisdomein 30, Maastricht, 6229 GT The Netherlands; 3 Research Centre for Autonomy and Participation for Persons with a Chronic Illness, Zuyd University of Applied Sciences, Nieuw Eyckholt 300, Heerlen, 6419DJ The Netherlands; 4 Academy for Nursing, Zuyd Health, Zuyd University of Applied Sciences, Nieuw Eyckholt 300, Heerlen, 6419DJ The Netherlands; 5 Department of Public Health and Primary Care (PHEG), Leiden University Medical Center, Leiden, the Netherlands; 6 Radboudumc Alzheimer Center and Department of Primary and Community Care, Radboud university medical center, Nijmegen, The Netherlands; 7 Living Lab in Ageing and Long-Term Care, Maastricht, The Netherlands; 8 Department of Research and Development, CIRO, Hornerheide 1, Horn, 6085 NM The Netherlands

**Keywords:** Advance care planning, End-of-life communication, Home care, Hospital, Nursing home, Nursing staff, Older people, Scoping review

## Abstract

**Background:**

Nursing staff is ideally positioned to play a central role in end-of-life communication as part of advance care planning for older people. However, this requires specific skills and competences. Only fragmented knowledge is available concerning important fundamentals in end-of-life communication performed by nursing staff.

**Objective:**

This review aimed to explore the fundamentals of end-of-life communication as part of advance care planning in the hospital, nursing home and home care setting, from the perspective of the nursing staff, the older person, and the family caregiver.

**Design:**

Scoping review.

**Methods:**

A literature search in PubMed, PsycINFO, CINAHL and Google (Scholar) was conducted on August 20, 2022. The search strategy followed the sequential steps as described in the Joanna Briggs Institute Manual. Peer-reviewed articles of empirical research and gray literature written in English or Dutch and published from 2010 containing fundamentals of end-of-life communication as part of advance care planning from the perspective of nursing staff, older people, and family caregivers in the hospital nursing home or home care setting were considered eligible for review.

**Results:**

Nine studies were included, and four themes were composed, reflecting 11 categories. Nursing staff attunes end-of-life communication to the values and needs of older people to approach the process in a person-centered manner. This approach requires additional fundamentals: building a relationship, assessing readiness, timing and methods to start the conversation, communication based on information needs, attention to family relationships, a professional attitude, improving communication skills, listening and non-verbal observation skills, and verbal communication skills.

**Conclusions:**

This review is the first to compile an overview of the fundamentals of end-of-life communication performed by nursing staff. Building a nursing staff-older-person relationship is the most important foundation for engaging in a person-centered end-of-life communication process. Knowing each other enables nursing staff to have a sense of older people’s readiness, determine the right timing to initiate an end-of-life conversation, identify specific needs, and accurately apply (non-)verbal observation skills. end-of-life communication is not a one-time conversation, but a complex process that takes time, effort, and genuine interest in each other.

**Supplementary Information:**

The online version contains supplementary material available at 10.1186/s12912-023-01523-2.

## Background

Advance care planning is a comprehensive, and ongoing [1] process that enables individuals to define goals and preferences for future medical treatment and care, discuss these goals and preferences with family and healthcare professionals, and record and adapt these preferences if appropriate [2]. End-of-life communication is an important component of advance care planning. “End-of-life communication as part of advance care planning” (hereafter called end-of-life communication) includes early and proactive formal (i.e., planned in advance) and informal (i.e., spontaneous) conversations between a person, family, and healthcare professional about future end-of-life care, the transition to the end-of-life phase, death, and dying from a holistic perspective [2–4].

Older people are frequently confronted with decisions concerning (life-sustaining) treatments and end-of-life care due to the development of illness and cognitive and physical limitations [5]. Moreover, older people report spending more time contemplating their end-of-life than younger people [6, 7]. End-of-life communication prepares older people and their family caregivers to undertake an active role in decision-making about future end-of-life care [8] and prevents them from receiving care that is not in line with their preferences (e.g., overtreatment, undertreatment, or unmet psychological and spiritual needs) [9].

Physicians are generally expected to take the lead in end-of-life communication with older people [10]. This can result in conversations that are more focused on the medical domain and do not include other domains, such as spirituality [10, 11]. Nursing staff is often more present and accessible for older people in hospital, home care, and nursing home settings compared to other healthcare professionals. They are trained to have a holistic view of older people’s care, develop a bond with older people and their family caregivers over time, and can naturally engage in formal and informal end-of-life communication as part of daily practice [12]. This makes them ideally positioned to play a central role in end-of-life communication with older people and their family caregivers [13]. However, nursing staff experiences challenges in end-of-life communication, such as feeling uncomfortable talking about death, a lack of training and guidance, and uncertainty regarding timing, roles, and responsibilities [14].

End-of-life communication tailored to an older person’s needs, requires specific skills and competencies [12]. To date, the available knowledge concerning important fundamentals in end-of-life communication performed by nursing staff is fragmented. Fundamentals refer to the important aspects involved in safe, effective, and high-quality end-of-life communication [15]. Knowing these fundamentals is necessary to educate nursing staff and to design and implement interventions that support nursing staff in improving end-of-life communication and taking a more leading role in advance care planning. Therefore, this scoping review addresses the following research question: *What are the fundamentals of* end-of-life *communication as part of* advance care planning *in the hospital, nursing home, and home care setting, from the perspective of nursing staff, the older person, and the family caregiver?*

## Methods

### Design

A scoping review method was used to explore the available literature describing the fundamentals of end-of-life communication from the perspective of nursing staff, older people, and family caregivers [16, 17]. A scoping review is a type of knowledge synthesis that uses a systematic and iterative approach to identify and synthesize an existing or emerging body of literature on a given topic [18]. This method was chosen to be able to explore and clarify the fundamentals of end-of-life communication, to determine the extent of research available on these fundamentals, and to identify gaps in the research knowledge base [19]. The Preferred Reporting Items for Systematic Reviews and Meta-analyses Extension for Scoping Reviews (PRISMA-ScR) were used to report the review (see Supplementary material table B: PRISMA ScR checklist) [17]. The review’s final search was carried out on August 20, 2022.

### Working group

An interprofessional working group (n = 16) was composed to verify the research method and (preliminary) results within the study. The group consisted of a patient representative, nursing staff of different levels working in the hospital, nursing home, and home care setting, members of a transmural palliative consultation team, a spiritual counselor, and other experts in palliative care, geriatric nursing care, and nursing education. This group gathered three times during the study and advised the research group on the composition of the search strings, the search strategy, the search results, and the thematic analysis.

### Search strategy and study selection process

The search strategy followed the sequential steps as described in the Joanna Briggs Institute Manual [19]. First, the databases PubMed, PsycINFO, and CINAHL and the search engines Google and Google Scholar were searched to identify relevant keywords and synonyms regarding the research subject in English and Dutch. Second, these terms were used to build search strings. Different combinations of the search terms were used to increase the sensitivity of the search strings and reduce the risk of missing relevant studies. An information specialist, the research group, and the working group helped define terminology and broaden definitions in the search strategy. An inclusive approach was used as generally recommended for scoping reviews [20]. A consensus on the search strings was reached with the research group.

Search terms represented the key subjects end-of-life communication and advance care planning and were combined using the Boolean operator “AND.” The search strings focused on keywords in titles and abstracts and were used in the databases PubMed, PsychINFO, and CINAHL (Supplementary material table A: Search strings). Titles and abstracts were screened based on the eligibility criteria in Table 1. The screening and selection of titles and abstracts were performed independently by the first author and cross-checked by the second and last author to increase the validity of the search. After screening and selecting titles and abstracts, eligible records were obtained as full texts. The screening and selection of the full-text articles were performed by the first, second, and last author. Any disagreements about the inclusion or exclusion of studies that arose between the reviewers were resolved through discussion or with an additional reviewer (fifth author) until a consensus was reached. The reference lists of eligible articles were hand-searched to identify other relevant articles. The reference lists of reviews were also searched for relevant references to original studies. Gray literature was searched using the search engines Google and Google Scholar. Experts in palliative care and advance care planning within the research group and the network of the research group and working group were asked if any relevant studies were missing in the composed selection.

**Table 1:** Inclusion and exclusion criteria

**Inclusion criteria**
• Peer-reviewed articles of empirical research. • Gray literature including reports, policy literature, dissertations, and white papers of relevant organizations (e.g., European Association for Palliative Care, World Health Organization, International Association for Hospice & Palliative Care) regarding palliative (nursing) care. • Containing fundamentals of end-of-life communication as part of advance care planning from the perspective of nursing staff (i.e., care assistants, certified nursing assistants, licensed vocational nurses, registered nurses, clinical nurse specialists, nurse practitioners), or (family caregivers of) older people in the hospital, nursing home or home care setting. o Studies that include healthcare professionals or clinicians in general will be included if nursing staff represents > 50% of the included professionals. o If older people are not specifically included in a study, the article will be included if the mean or median age of the included people is at least 65 years.
**Exclusion criteria**
• Not written in English or Dutch. • Published before 2010. • Studies regarding advance care planning that do not meet the definition: a comprehensive, and ongoing process that enables individuals to define goals and preferences for future medical treatment and care, discuss these goals and preferences with family and healthcare professionals, and record and adapt these preferences if appropriate. • Studies regarding end-of-life communication as part of advance care planning that do not meet the definition: early and proactive formal (i.e., predetermined) and informal (i.e., spontaneous) conversations between a person, family, and healthcare professional about future end-of-life care, the transition to the end-of-life phase, death, and dying from a holistic perspective. For example, conversations at the end-of-life about current care or conversations with the family caregiver about the older person while the older person is not present.

### Data synthesis and analysis

All data from the included papers were extracted by the first author and cross-checked by the second and last author. General information about the included studies was extracted using a data extraction form, including the names of the authors, date of publication, country, design, the aim of the study, setting, sample, and research method. Data synthesis was applied following the Johanna Briggs Institute Manual [19]. The complete “Results” or “Findings” sections of the included qualitative studies (including quotes) were extracted for the analysis. The “Results” sections of quantitative studies were extracted and summarized by the first author. The extracted qualitative data and the summarized quantitative data were both analyzed using a thematic analysis approach following the principles of Braun and Clarke [21]. The first author read all the articles and followed the iterative process of open, axial, and selective coding to identify relevant themes and categories. After becoming familiar with the qualitative data, initial codes were identified, the data of the first three included studies were coded after which the generated codes were reviewed, merged, and grouped into categories if relevant. Hereafter, the data of the next three studies were coded and reviewed. After this step, the first few themes were created. Then, the data of the last studies were coded, initial themes were reviewed, and more themes were created when necessary. From this point, the aggregated meaning of the themes and categories was discussed in the research group and working group until consensus was reached.

The third and fourth author applied the same approach to each perform an independent thematic analysis on, respectively, 22% (n = 2 articles focusing on the nursing staff perspective) and 33% (n = 3 articles focusing on the older person perspective) of the included articles. The analyses were compared and discussed until a consensus was reached between the first, third, and fourth author. The second and last author cross-checked the analysis and the identified themes and categories. Themes and categories were defined and described for further analysis and reporting of the results by the first author in continuous consultation and alignment with the working group and the second, fifth, sixth and last author to eventually reach a consensus. Data analysis was performed using Atlas.ti (version 9.1.3).

## Results

### Literature search

**Fig. 1:**
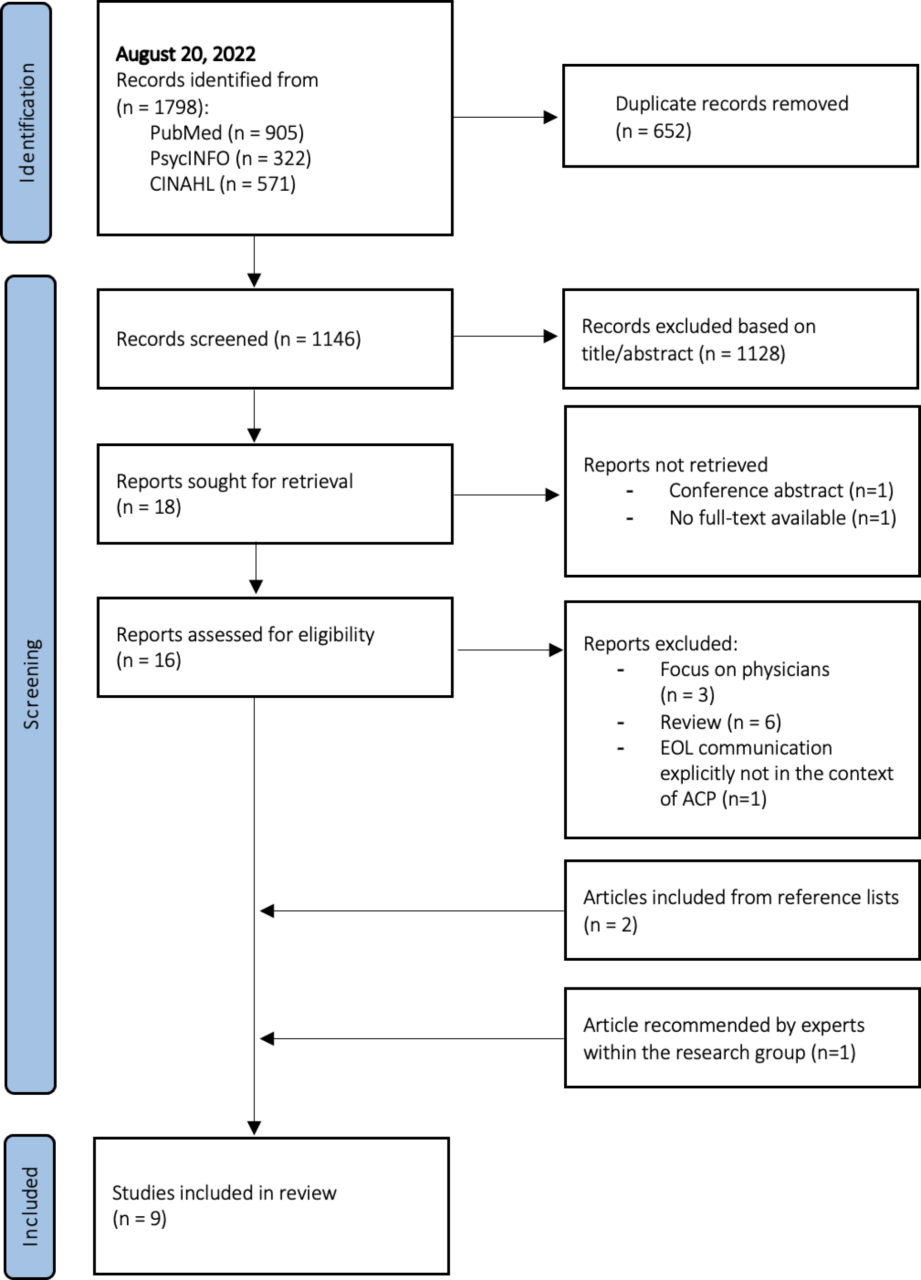
Flow diagram of the selection process

After removing duplicates, the search resulted in a total of 1146 unique publications. A total of nine articles representing nine studies remained after selection and were included (Fig. 1). In the gray literature, no eligible reports were found.

### Data extraction outcomes

The studies originated from seven countries (the United States, England, Germany, Norway, Australia, Japan, and Canada) and were published between 2010 and 2020 (Table 2). Six studies showed the nursing staff perspective, two studies showed the older person’s perspective, and one study described both. Two studies also described the family caregiver perspective, but the data described from this perspective was very limited. The results primarily focus on the nursing staff and older person perspective. Six studies used a qualitative approach: focus groups (n = 3), individual interviews (n = 2), and an exploratory case study (n = 1). Three quantitative cross-sectional studies were included (two surveys and one with structured interviews). Several studies were conducted within multiple settings, including the hospital (n = 8), nursing home (n = 3), and home care (n = 4).

**Table 2:** Included studies

Author (year) *Country*	Research method and design	Aim	Setting	Sample (n)	Nursing staff perspective	Older person perspective
**Almack et al.** (2012) *England*	Exploratory case study Qualitative	To explore the factors influencing if, when and how EOL communication takes place between healthcare professionals, older people, and family caregivers from the perspectives of all parties involved and how preferences are discussed and recorded.	Hospital Home care Nursing home GP practice service	Older people with and without a cancer diagnosis (n = 18) and family caregivers (n = 11) and healthcare professionals (nursing staff n = 8, 53%)	X	X
**Groebe et al.** (2019) *Germany*	Focus group study Qualitative	To give insights into specific requirements and conditions for EOL conversations in various EOL care settings.	Hospital Home care Nursing home	Volunteers and nursing staff (n = 11, 61%) and psychosocial care	X	
**Hjorth et al.** (2018) *Norway*	Focus group study Qualitative	To explore the needs and preferences of older people with pulmonary diseases regarding ACP to prepare for the introduction of ACP in Norwegian hospitals.	Hospital	Older people receiving treatment for advanced lung cancer, COPD, or lung fibrosis (n = 13)		X
**Isaacson et al.** (2018) *United States*	Interview study Qualitative	To understand the shared communication practices of rural and urban, hospice/palliative care nursing staff when engaging older people with a terminal diagnosis and their family caregivers about decision-making.	Hospital Home care Nursing home Hospice house	Hospice/palliative care nursing staff (n = 10)	X	
**Kerr et al.** (2019) *Australia*	Focus group study Qualitative	To identify challenges for nursing staff when communicating with people who have life limiting illness, and their family caregivers.	Hospital	Nursing staff (n = 39)	X	
**Kimura et al.** (2020) *Japan*	Cross-sectional survey study Quantitative	To identify barriers to EOL discussions with older people with an advanced cancer diagnosis and their family caregivers as perceived by oncologists, nursing staff, and medical social workers, as well as to clarify effective strategies to facilitate EOL discussions.	Hospital	Physicians, nursing staff (n = 993 (53%)*), and medical social workers	X	
**Reinke et al.** (2010a) *United States*	Interview study Qualitative	To examine nursing staff perspectives on meeting older people’s needs for hope and illness information and to offer insights for interventions designed to improve EOL communication for older people and their family caregivers.	Outpatient setting	Nursing staff (n = 22)	X	
**Reinke et al.** (2010b) *United States*	Cross-sectional survey study Quantitative	To identify nursing staff perspectives on nursing skills that are important yet under-utilized in EOL care.	Hospital Hospice (in-patient, outpatient, and home care) Outpatient clinic	Nursing staff (n = 717)	X	
**You et al.** (2014) *Canada*	Structured interview study Quantitative	To determine which of the guideline-recommended fundamentals to include in discussions about goals of care are most important to older people with serious illness and their family caregivers.	Hospital	Older people (n = 233) and their family caregivers (n = 205)		X

ACP, advance care planning

COPD, chronic obstructive pulmonary disease

EOL, end-of-life

GP, General Practitioner

* Nursing staff results described separately

### Thematic analysis and interpretation

Four themes were composed, reflecting 11 categories and 243 codes: “person-centered approach,” “preparing for end-of-life communication,” “carrying out end-of-life communication,” and “professional attitude and required skills” (see Table 3 and Supplementary material table D: Codebook). The themes represent areas within the end-of-life communication process, while the categories represent the fundamentals of the end-of-life communication process.

**Table 3:** Contribution to identified themes and categories

Author (year)	Contribution to identified themes and categories																					
	*Person-centered approach*		*Preparing for EOL communication*								*Carrying out EOL communication*				*Professional attitude and required skills*							
			Building a relationship with the conversation partner		Assessing the readiness of the older person		Timing and methods to start EOL communication		Practical requirements when preparing the EOL conversation		EOL communication based on information needs		Attention to the family relationship		Professional attitude		Improving EOL communication skills		Listening and non-verbal observation skills		Verbal communication skills	
Perspective	Nursing staff	Older person	Nursing staff	Older person	Nursing staff	Older person	Nursing staff	Older person	Nursing staff	Older person	Nursing staff	Older person	Nursing staff	Older person	Nursing staff	Older person	Nursing staff	Older person	Nursing staff	Older person	Nursing staff	Older person
Almack et al. (2012)	X		X		X	X	X				X						X		X		X	
Groebe et al. (2019)	X		X		X		X		X		X		X		X		X		X		X	
Hjorth et al. (2018)		X		X		X		X				X		X								
Isaacson et al. (2018)	X		X		X		X		X		X		X		X		X		X		X	
Kerr et al. (2019)	X		X		X		X		X		X		X		X		X		X		X	
Kimura et al. (2020)	X								X				X									
Reinke et al. (2010a)	X		X		X				X		X								X			
Reinke et al. (2010b)	X				X				X		X		X		X		X		X			
You et al. (2014)												X										

EOL, end-of-life

### Theme 1: person-centered approach

A person-centered approach is central to the end-of-life communication process. It is the starting point for each fundamental. Using this approach, the nursing staff adjusts the end-of-life communication process to the older person’s individual values and needs. Nursing staff finds it important to advocate for the older person [12], to maintain the older person’s dignity, 22] and to acknowledge that the feelings and thoughts of the older person are central to end-of-life communication [22–24]. Older people correspondingly prefer an individual approach to end-of-life communication in which their values and needs are respected [25].

According to nursing staff, the older person should be in the lead with end-of-life communication [12, 22, 23, 26] and shared decision-making should be applied [12]. Older people want to be asked about their needs for communicating and planning and to make their own decisions in line with their preferences [25]. Shared decision-making can ensure that older people’s preferences, needs, and values guide decisions on future care [12].

### Theme 2: preparing for end-of-life communication

#### Building a relationship with the conversation partner

According to nursing staff and older people, the foundation of a “good” end-of-life communication process is a strong relationship between nursing staff, the older person, and the family caregiver. For nursing staff, this relationship assumes trust, 22, 23, 26, 27] safety, 22] support, 22] continuity, 22] and getting to know the older person [23, 27] as the most important goals. Although nursing staff is ideally positioned to achieve this, 12] anyone could be a good conversation partner since it depends more on the professional relationship with the older person than on the profession itself [25, 26]. Older people prefer a conversation partner of a certain sex, with whom they have a reasonable age difference, 25] who is skilled in communication, and someone who makes them feel safe, understands them and is knowledgeable about them, the disease, and the last phase of life [25].

#### Assessing the readiness of the older person

According to nursing staff and older people, it is vital to assess the older person’s readiness before initiating end-of-life communication [12, 22–24, 26, 27], and to not force the older person to engage in end-of-life communication [25–27]. Older people may not want to think too far ahead [27] or may not feel prepared to engage in end-of-life communication [25]. Nursing staff indicates that variations in older people’s coping, awareness, and understanding regarding their illness can influence readiness [12, 23, 24, 26, 27]. Older people can also be frustrated and angry, 12] cope with taboos regarding death and dying, 26] or not consider themselves in need of participating in end-of-life communication [27]. However, these attitudes can change over time [26]. Therefore, it is necessary for nursing staff to “re-assess” the older person’s readiness regularly [22]. Nursing staff can use listening- and non-verbal observation skills to (re-)assess readiness [24]. Older people have indicated that end-of-life communication should be performed while being cognitively sound, and when distressing symptoms (e.g., dyspnea or pain) are well treated [25]. Furthermore, because older people sometimes struggle with knowing what to ask during end-of-life conversations, they prefer to be alerted or receive an introduction before the conversation to prepare and invite other people to participate [25].

#### Timing and methods to start end-of-life communication

Finding the right timing to initiate end-of-life communication is considered essential [26], but difficult for nursing staff [12]. It requires preparatory work, such as building a relationship and assessing readiness [27]. Nursing staff must be alert to communication cues (e.g., “I might not even make it home,” “I cannot bounce back from this,” “I wish I were dead”) to find an opening to start conversations at the right moment [12, 22, 27]. Nursing staff uses listening and non-verbal observation skills to find these cues [22] and use their intuition to “feel” and “know” the right moment [12, 27]. This intuition can be supported by the relationship between nursing staff and the older person. Nursing staff can prudently use prompts, inviting phrases, 22] and appropriate openings, such as trigger questions or easily accessible topics, 26] to create an opening to start the conversation [26]. Hjorth et al. [25] found that older people prefer nursing staff to initiate end-of-life conversations instead of having to initiate the conversations themselves. In addition, they prefer early end-of-life conversations at different turning points in their disease trajectory, including at the time of diagnosis and when anxiety increases [25].

#### Practical requirements when preparing the end-of-life conversation

When the right moment for an end-of-life conversation is determined by the nursing staff, the practical aspects of the conversation should be prepared. Conversations must occur in a room that offers privacy [28] in a safe and supportive environment in which an older person feels comfortable [22, 23]. Nursing staff should be completely present [22, 23] and should not be disturbed by colleagues or other tasks or time restraints [12, 22, 24, 26, 28]. Nursing staff should also sit on the same level instead of standing over the older person [22]. Furthermore, nursing staff should be informed about what other involved professionals have already discussed with the older person [23, 24].

### Theme 3: carrying out end-of-life communication

#### End-of-life communication based on information needs

Assessing information needs is considered important by nursing staff and older people in every end-of-life conversation [12, 25, 29]. Nursing staff assess these needs based on the degree of awareness and knowledge of the older person and family caregivers related to the disease [12]. Carefully assessing the older person’s information needs can prevent nursing staff from making false assumptions, 23] provide support by providing consistent and accurate information, 12, 23, 24] and give older people sufficient space to discuss topics that are important to them [22]. According to nursing staff, these topics can consist of, for example, symptom and pain control, 23, 24] hope, 23, 27] and what gives life meaning [23]. Older people prefer discussing topics such as treatment and side effects, 25] fears and concerns, 29] and existential and spiritual issues [26]. Older people want nursing staff to approach end-of-life conversations carefully and delicately and desire the conversations to be safe, respectful, transparent, and honest [25].

#### Attention to the family relationship

As family structures become increasingly complex, 12] paying attention to the family relationship in the end-of-life communication process is important, according to nursing staff and older people [12, 22, 24–26, 28]. Nursing staff advises paying attention to family relationships in order to align their wishes with those of the older person [22, 24, 28]. Older people value a good family relationship and prefer having family around who are knowledgeable, supportive, and caring [25]. They usually want to be open about future end-of-life issues with family members but express the need to be assisted by nursing staff when doing so [25]. Nursing staff also requests that older people and their family members should express feelings, talk openly, and listen to each other during end-of-life communication [22, 26]. This can prevent them from needing to repeat information for family members and can help them avoid difficult situations in which they are, for example, asked by family members not to disclose specific information to the older person [12].

### Theme 4: professional attitude and required skills

#### Professional attitude

It is important that nursing staff adopts a professional attitude in the end-of-life communication process [12, 22, 24, 26]. Nursing staff should be supportive, 26] calm, 22] compassionate, 22] confident, 12] gentle, 26] and emphatic [12, 22]. Being genuinely interested, 26] comfortable in silence [22] and talking about dying, 24] respectful towards cultural and religious beliefs, 24] and nursing staff being self-aware of their nonverbal cues, emotional response, and role [12, 22, 26] contributes to the expression of this attitude. Nursing staff should not be judgmental [22, 24, 26] nor try to change perceptions of the older person [22]. This professional attitude contributes to the development of a strong relationship with the older person and effective communication.

#### Improving end-of-life communication skills

Nursing staff often feels unskilled [12, 24] and insecure as they sometimes do not exactly know what to do when, for example, end-of-life conversations become difficult and they do not have a toolkit to rely on [12]. This can result in feeling uncomfortable talking about death and avoiding end-of-life communication [24]. To overcome these challenges and be able to engage in high-quality end-of-life communication, nursing staff needs sufficient education and training in how to conduct end-of-life conversations and how to deal with conversations about death and dying [12, 22, 24, 26, 27]. In addition, nursing staff needs opportunities to develop practical experience in advanced end-of-life communication skills [12, 27].

#### Listening and non-verbal observation skills

Attentive listening and non-verbal observation skills support nursing staff in guiding end-of-life communication. Nursing staff should be attentive to the subtle language older people use [22, 26] so they can “read” them through eye contact [22] and nonverbal signs [12, 22]. Skills to read and express observations support nursing staff in assessing readiness, initiating conversations at the right moment, navigating conversations [12, 22, 26], making older people feel heard and seen [22], and stimulating older people to express feelings and reflect on their feelings [22]. It can ensure that all voices are heard [22], evaluate older people’s level of awareness and understanding of the conversation [27], and ensure that the tempo of the conversation as set by the older person and family caregiver is respected [22]. These skills also contribute to the nursing staff’s ability to get to know the older person’s way of communicating (as part of building a relationship) and thus their ability to apply listening and non-verbal observation skills correctly. Isaacson et al. [22] refer to this as “learning the older person’s rhythm.” This rhythm can be specific to how an older person expresses physical, spiritual, or emotional concerns [22].

#### Verbal communication skills

Nursing staff must be able to assess which words and verbal communication skills are appropriate in end-of-life communication [12, 22, 26, 27]. For example, nursing staff prefers using the word “uncomfortable” over the word “pain” when assessing older people’s symptoms. This stimulates older people to share more information [22]. Moreover, sentences such as “Is there something you would like to do while you are still physically able?” are more appropriate compared to talking about “goals of care” [26]. Nursing staff must approach end-of-life communication in an open, detailed, narrative [26], and understandable [22] way. Combining this approach with the repetition of messages in different ways can be valuable to assure the older person understands the information being said [22]. Wishes regarding nursing staff’s verbal communication are individual.

To support nursing staff’s verbal end-of-life communication skills, several tools are available. However, there is ambiguity around the usefulness of these tools in clinical practice [26]. Tools support open and detailed communication and are considered important to clarify older people’s attitudes [26]. However, the use of tools can also result in a lack of individuality and is considered counterintuitive to nursing staff’s professional attitude. Tools can contain direct questions, which are considered potentially harmful to the quality and contemplation of end-of-life communication [26].

## Discussion

In this scoping review, the available literature regarding the fundamentals of end-of-life communication from the perspective of the nursing staff, older person, and family caregiver was explored. Four themes emerged. First, “having a person-centered approach” is considered central throughout the end-of-life communication process. Second, “preparing for end-of-life communication,” in which building a relationship between the nursing staff and the older person, assessing the readiness of the older person, timing and initiation of end-of-life communication, and practical requirements are fundamental. Third, “carrying out end-of-life communication” was identified, which focuses on the information needs of older people in end-of-life communication and pays attention to the older person-family relationship. Fourth, “professional attitude and required skills” was found, which included improving end-of-life communication skills, professional attitude, listening and (non-)verbal communication and observation skills as central aspects.

Nursing staff attunes end-of-life communication to the values and needs of older people to approach the process in a person-centered manner. To be able to strive for this approach requires the application of the identified fundamentals in this review. Especially building a strong nursing staff-older-person relationship, which is considered the most important fundament of person-centered end-of-life communication and the basis of nursing care, requires a specific training process [30]. A strong relationship enables close, constructive, and effective communication on an equal level between the nursing staff and the older person [31]. This corresponds to the Theory of Human Communication, which estimates that the factual level of communication (e.g., information, data, and figures) only constitutes 10–20% of communication. The remaining 80–90% are, often unconsciously, on a deeper relationship level [32]. Knowing each other enables nursing staff to assess and sometimes intuitively sense older people’s readiness, the right timing to initiate an end-of-life conversation, and specific needs, and to accurately apply listening and (non-)verbal observation skills.

The results of this review emphasize that end-of-life communication is not a one-time conversation but a complex process that takes time, effort, and genuine interest in each other. When nursing staff is aware of the importance of a good relationship with older people, this helps them apply a tailored approach to end-of-life communication according to the fundamentals identified in this review. According to the “Fundamentals of Care” framework, the development of trusting therapeutic relationships is also considered very important in nursing care in general [15]. The framework stresses the need to integrate people’s different fundamental needs, which are mediated through the nursing staff’s relational actions, like active listening and being empathic [15]. Mostly, informal conversations can contribute to this process. Informal conversations enable nursing staff to spend a lot of time with the older person, make communicating accessible, and build a trusting relationship. In addition, nursing staff’s holistic view of older people’s care enables them to build deep and strong relationships in which the older person is seen as a whole [33].

Relationships help to shape the way nursing staff and older people perceive and interact with each other [34]. This can also contribute to older people’s feeling of readiness to engage in end-of-life communication. Older people should feel at ease and ready to discuss sensitive topics related to the end-of-life and with the nursing staff. A potential threat to this readiness could be the nursing staff not feeling ready to engage in end-of-life communication. This can lead to the avoidance of and insufficiency of end-of-life communication [35, 36]. While many studies describe the importance of readiness in (older) people as an important fundamental for end-of-life communication, nursing staff’s and family caregivers’ readiness is rarely mentioned [36–38]. This is also evident from the results of this review. Nevertheless, before engaging in the end-of-life communication process, every person involved should consider and be aware of their thoughts and values regarding death and dying and which potential factors might influence those. Especially nursing staff should be aware of their own thoughts and values regarding the end-of-life to prevent them from negatively influencing the end-of-life communication process, and to grow in the process itself.

End-of-life communication with older people should be initiated at an early stage, according to many studies. Remarkably, no studies included in this review solely focus on end-of-life communication in the home care setting, while initiating end-of-life communication in the hospital setting is often considered “too late.” In the home care setting, older people can be prepared for (future) decisions related to the end-of-life more easily, and before serious physical and cognitive limitations develop [39]. In addition, because of the continuity of care in the home care setting, more time can be spent building a strong nursing staff-older-person relationship. Early initiation of end-of-life communication in the home care setting can also contribute to the continuity of care between different healthcare settings, for example, when earlier discussed information between nursing staff and an older person is registered and shared appropriately between the different healthcare settings [39]. When nursing staff in different healthcare settings is aware of this information, building relationships might also become more convenient.

### Strengths and limitations

To the authors’ best knowledge, this study is the first to compile an overview of the fundamentals of end-of-life communication with older people specifically performed by nursing staff (and their mutual interrelationships). In addition, the scoping review followed the sequential steps as described in the Johanna Briggs Institute Manual, with at least two researchers involved in each step. Furthermore, an interprofessional working group was involved in all phases of the study. This has contributed to the optimization of the search strategy, the practical verification of the results, and therefore the reliability of the study. Besides these strengths, some limitations are also worth mentioning. First, only nine studies could be included in this review. This might be a result of the limited evidence but might also be due to the lack of a clear definition of end-of-life communication in studies on this subject. This is a general problem in studies on end-of-life communication [40, 41], and was even more evident in this review. The focus on end-of-life communication as part of advance care planning is often not defined explicitly, but only written between the lines, which has made the selection process for the review complex. Second, while the studies included in this review covered a wide range of end-of-life communication fundamentals, the in-depth understanding and practical translations of these fundamentals were frequently lacking. Third, the studies included in this review primarily focus on the hospital setting and the nursing staff perspective. This is an often-mentioned limitation in palliative care research [42] and has resulted in an underrepresentation of the home care and nursing home settings in the results of this review, and five of the eleven defined categories are not described from the perspective of the older person. Moreover, the family caregivers’ perspective was only mentioned in two of the included studies and the data described from this perspective were limited. The family caregiver perspective therefore was insufficiently described in this review. Fourth, the articles included in this review focus on formally planned end-of-life communication, while informal end-of-life communication could greatly support building a nursing staff-older-person relationship and therefore end-of-life communication in general. Fifth, although the included studies were performed in seven different countries, these studies showed similar results. This is noteworthy as the delivery of palliative care can vary greatly between countries based on, for example, socioeconomic conditions and cultural issues [43]. Sixth, thesis databases and official websites were not searched as part of this review. This may have limited the search of this review.

### Future research

The present scoping review indicates areas for future studies. First, some fundamentals lack a deep understanding and practical application. For example, it is unclear how to express and measure nursing staff’s adaptation of a professional attitude (e.g., being supportive, calm, and compassionate) in clinical practice. More qualitative research concerning in-depth descriptions of the fundamentals of end-of-life communication from the perspective of nursing staff, older people, and family caregivers is necessary to increase the evidence base on this subject. In addition, these studies should focus on diverse healthcare settings (i.e., home care and nursing homes) to increase transferability. Second, qualitative research on formal and informal end-of-life communication between nursing staff and older people (and family caregivers) is necessary to explore the dynamics between the fundamentals and to add missing fundamentals to the provided overview. These strategies collectively contribute to developing resources to educate nursing staff in conducting and taking a central role in end-of-life communication and in designing and implementing interventions to support nursing staff in improving end-of-life communication.

## Conclusions

The identified fundamentals of end-of-life communication can guide nursing staff in applying a tailored approach to end-of-life communication. end-of-life communication is not a one-time conversation but a complex process that takes time, effort, and genuine interest in each other. Building a nursing staff-older-person relationship is considered the most important foundation for nursing staff and older people to engage in a person-centered end-of-life communication process. More qualitative research is needed to improve practical translations and an in-depth understanding of the fundamentals, particularly from the perspective of older people and family caregivers, as well as in home care and nursing home settings.

### Electronic supplementary material

Below is the link to the electronic supplementary material.

**Supplementary Material 1: Table A**. Search strings

**Supplementary Material 2: Table B**. PRISMA-ScR Checklist

**Supplementary Material 3: Table C**. Included study results and contribution per theme

**Supplementary Material 4: Table D**. Codebook

## Data Availability

The datasets used and/or analyzed during the current study available from the corresponding author on reasonable request.

## References

[1] Sinuff T, Dodek P, You JJ, Barwich D, Tayler C, Downar J (2015). Improving end-of-life communication and decision making: the development of a conceptual framework and quality indicators. J Pain Symptom Manag.

[2] Rietjens JA, Sudore RL, Connolly M, van Delden JJ, Drickamer MA, Droger M (2017). Definition and recommendations for advance care planning: an international consensus supported by the European Association for Palliative Care. Lancet Oncol.

[3] Schüttengruber G, Großschädl F, Lohrmann C. A Consensus definition of end of life from an International and Interdisciplinary Perspective: a Delphi Panel Study. J Palliat Med. 2022. 10.1089/jpm.2022.0030 35549439

[4] Sudore RL, Lum HD, You JJ, Hanson LC, Meier DE, Pantilat SZ (2017). Defining advance care planning for adults: a consensus definition from a multidisciplinary Delphi panel. J Pain Symptom Manag.

[5] Hopkins SA, Bentley A, Phillips V, Barclay S (2020). Advance care plans and hospitalized frail older adults: a systematic review. BMJ Supportive & Palliative Care.

[6] Lockhart LK, Bookwala J, Fagerlin A, Coppola KM, Ditto PH, Danks JH (2001). Older adults’ attitudes toward death: links to perceptions of health and concerns about end-of-life issues. OMEGA-Journal of Death and Dying.

[7] Mohammadpour A, Sadeghmoghadam L, Shareinia H, Jahani S, Amiri F (2018). Investigating the role of perception of aging and associated factors in death anxiety among the elderly. Clin Interv Aging.

[8] Rietze L, Stajduhar K (2015). Registered nurses’ involvement in advance care planning: an integrative review. Int J Palliat Nurs.

[9] Detering KM, Hancock AD, Reade MC, Silvester W. The impact of advance care planning on end of life care in elderly patients: randomised controlled trial. BMJ. 2010;340. 10.1136/bmj.c1345 PMC2844949 20332506

[10] Izumi S (2017). Advance care planning: the nurse’s role. AJN the American Journal of Nursing.

[11] Dixon J, Knapp M (2018). Whose job? The staffing of advance care planning support in twelve international healthcare organizations: a qualitative interview study. BMC Palliat care.

[12] Kerr D, Milnes S, Ammentorp J, McKie C, Dunning T, Ostaszkiewicz J (2020). Challenges for nurses when communicating with people who have life-limiting illness and their families: a focus group study. J Clin Nurs.

[13] Phillips J, Johnston B, McIlfatrick S. Valuing palliative care nursing and extending the reach. SAGE Publications Sage UK: London, England;; 2020. pp. 157–9. 10.1177/0269216319900083 32009566

[14] Hemsley B, Meredith J, Bryant L, Wilson NJ, Higgins I, Georgiou A (2019). An integrative review of stakeholder views on advance care directives (ACD): barriers and facilitators to initiation, documentation, storage, and implementation. Patient Educ Couns.

[15] Kitson A, Conroy T, Kuluski K, Locock L, Lyons R. Reclaiming and redefining the Fundamentals of Care: Nursing’s response to meeting patients’ basic human needs. 2013.

[16] Munn Z, Peters MD, Stern C, Tufanaru C, McArthur A, Aromataris E (2018). Systematic review or scoping review? Guidance for authors when choosing between a systematic or scoping review approach. BMC Med Res Methodol.

[17] Tricco AC, Lillie E, Zarin W, O’Brien KK, Colquhoun H, Levac D (2018). PRISMA extension for scoping reviews (PRISMA-ScR): checklist and explanation. Ann Intern Med.

[18] Mak S, Thomas A (2022). Steps for conducting a scoping review. J Graduate Med Educ.

[19] Peters M, Godfrey C, McInerney P, Soares C, Khalil H, Parker D. The Joanna Briggs Institute reviewers’ manual 2015: methodology for JBI scoping reviews. 2015.

[20] Arksey H, O’Malley L (2005). Scoping studies: towards a methodological framework. Int J Soc Res Methodol.

[21] Braun V, Clarke V (2019). Reflecting on reflexive thematic analysis. Qualitative Res Sport Exerc Health.

[22] Isaacson MJ, Minton ME (2018). End-of-life communication: nurses cocreating the closing composition with patients and families. ANS Adv Nurs Sci.

[23] Reinke LF, Shannon SE, Engelberg RA, Young JP, Curtis JR (2010). Supporting hope and prognostic information: nurses’ perspectives on their role when patients have life-limiting prognoses. J Pain Symptom Manag.

[24] Reinke LF, Shannon SE, Engelberg R, Dotolo D, Silvestri GA, Curtis JR (2010). Nurses’ identification of important yet under-utilized end-of-life care skills for patients with life-limiting or terminal illnesses. J Palliat Med.

[25] Hjorth NE, Haugen DF, Schaufel MA. Advance care planning in life-threatening pulmonary disease: a focus group study. ERJ Open Res. 2018;4(2). 10.1183/23120541.00101-2017 PMC5958273 29796390

[26] Groebe B, Rietz C, Voltz R, Strupp J (2019). How to talk about attitudes toward the end of life: a qualitative study. Am J Hosp Palliat Care.

[27] Almack K, Cox K, Moghaddam N, Pollock K, Seymour J (2012). After you: conversations between patients and healthcare professionals in planning for end of life care. BMC Palliat care.

[28] Kimura Y, Hosoya M, Toju K, Shimizu C, Morita T (2020). Barriers to end-of-life discussion with advanced cancer patient as perceived by oncologists, certified/specialized nurses in cancer nursing and medical social workers. Jpn J Clin Oncol.

[29] You JJ, Dodek P, Lamontagne F, Downar J, Sinuff T, Jiang X (2014). What really matters in end-of-life discussions? Perspectives of patients in hospital with serious illness and their families. CMAJ.

[30] Allande-Cussó R, Fernández-García E, Porcel-Gálvez AM (2022). Defining and characterising the nurse–patient relationship: a concept analysis. Nurs Ethics.

[31] Ryan T (2022). Facilitators of person and relationship-centred care in nursing. Nurs Open.

[32] Watzlawick P, Bavelas JB, Jackson DD. Pragmatics of human communication: a study of interactional patterns, pathologies and paradoxes. WW Norton & Company; 2011.

[33] Papathanasiou I, Sklavou M, Kourkouta L (2013). Holistic nursing care: theories and perspectives. Am J Nurs Sci.

[34] Kiernan E (2015). Building professional nursing communication.

[35] Clare E, Elander J, Baraniak A. How healthcare providers’ own death anxiety influences their communication with patients in end-of-life care: A thematic analysis. Death Stud. 2020:1–8. 10.1080/07481187.2020.1837297 33108977

[36] Nia HS, Lehto RH, Ebadi A, Peyrovi H (2016). Death anxiety among nurses and health care professionals: a review article. Int J Community Based Nurs Midwifery.

[37] Boyd D, Merkh K, Rutledge DN, Randall V, editors. Nurses’ perceptions and experiences with end-of-life communication and care. Oncology nursing forum; 2011. 10.1188/11.ONF.E229-E239 21531673

[38] Malone LD, Anderson J, Croxon L (2016). Are newly graduated nurses ready to deal with death and dying?-A literature review. Nurs Palliat Care.

[39] Howard M, Bernard C, Tan A, Slaven M, Klein D, Heyland DK (2015). Advance care planning: let’s start sooner. Can Fam Physician.

[40] Chen W, Chung JOK, Lam KKW, Molassiotis A. End-of-life communication strategies for healthcare professionals: a scoping review. Palliat Med. 2022. 02692163221133670. 10.1177/02692163221133670 36349371

[41] Brighton LJ, Koffman J, Hawkins A, McDonald C, O’Brien S, Robinson V (2017). A systematic review of end-of-life care communication skills training for generalist palliative care providers: research quality and reporting guidance. J Pain Symptom Manag.

[42] Bolt SR, van der Steen JT, Mujezinović I, Janssen DJ, Schols JM, Zwakhalen SM (2021). Practical nursing recommendations for palliative care for people with dementia living in long-term care facilities during the COVID-19 pandemic: a rapid scoping review. Int J Nurs Stud.

[43] Zhou K, Fu J. Evolution of Oncology and Palliative Nursing in Meeting the Changing Landscape of Cancer Care. Journal of Healthcare Engineering. 2022;2022. 10.1155/2023/9852613 PMC10232186 37266194

