# Supplementary material for: Fundamentals of end-of-life communication as part of advance care planning from the perspective of nursing staff, older people, and family caregivers: a scoping review
Source: BMC Nurs. 2023 Oct 6;22:363. doi: 10.1186/s12912-023-01523-2 (PMC10559445; doi:10.1186/s12912-023-01523-2)
Supplement: Supplementary file 1 — Supplementary Material 1: Table A. Search strings [file 12912_2023_1523_MOESM1_ESM.docx]

**Supplementary table A - “Search strings”**

| **Database: PubMed** | **Filters** | **Results** |
| --- | --- | --- |
| ((end of life communicat*[Title/Abstract]) OR (end of life convers*[Title/Abstract]) OR (end of life discuss*[Title/Abstract]) OR (end of life decision-mak*[Title/Abstract]) OR (end of life talk*[Title/Abstract]) OR (choices related to end of life[Title/Abstract]) OR (choices related to death[Title/Abstract]) OR (choices related to dying[Title/Abstract]) OR (choices about end of life[Title/Abstract]) OR (choices about dying[Title/Abstract]) OR (choices about death[Title/Abstract]) OR (communication about end of life[Title/Abstract]) OR (communication about death[Title/Abstract]) OR (communication about dying[Title/Abstract]) OR (death dying and communication[Title/Abstract]) OR (communication strategies about end of life[Title/Abstract]) OR (communication strategies about death[Title/Abstract]) OR (communication strategies about dying[Title/Abstract])) **AND** ((advance care plan*[Title/Abstract]) OR (advance care directive*[Title/Abstract]) OR (advance healthcare plan*[Title/Abstract]) OR (advance health care plan*[Title/Abstract]) OR (advance healthcare directive*[Title/Abstract]) OR (advance health care directive*[Title/Abstract]) OR (advance directive*[Title/Abstract]) OR (living will*[Title/Abstract]) OR (ACP[Title/Abstract]) OR ("Advance Care Planning"[MeSH Terms]) OR (end of life care[Title/Abstract]) OR (end of life plan*[Title/Abstract]) OR (end of life healthcare[Title/Abstract]) OR (end of life health care[Title/Abstract]) OR (proactive health care[Title/Abstract]) OR (proactive healthcare[Title/Abstract]) OR (proactive care[Title/Abstract]) OR (goals of care[Title/Abstract])) | Pub. date from 2010 – 2022 | 905 |

| **Database: PsycINFO** | **Filters** | **Results** |
| --- | --- | --- |
| ( TI ( "end of life communicat*" OR "end of life convers*" OR "end of life discuss*" OR "end of life decision-mak*" OR "end of life talk*" OR “choices related to end of life” OR “choices related to death” OR “choices related to dying” OR “choices about end of life” OR “choices about dying” OR “choices about death” OR “communication about end of life” OR “communication about death” “communication about dying” OR “death and dying communication” OR “communication strategies about end of life” OR “communication strategies about death” OR “communication strategies about dying” ) OR AB ( "end of life communicat*" OR "end of life convers*" OR "end of life discuss*" OR "end of life decision-mak*" OR "end of life talk*" OR “choices related to end of life” OR “choices related to death” OR “choices related to dying” OR “choices about end of life” OR “choices about dying” OR “choices about death” OR “communication about end of life” OR “communication about death” “communication about dying” OR “death and dying communication” OR “communication strategies about end of life” OR “communication strategies about death” OR “communication strategies about dying”) ) **AND** ( TI ( "advance care plan*" OR "advance care directive*" OR "advance healthcare plan*" OR "advance health care plan*" OR "advance healthcare directive*" OR "advance health care directive*" OR "advance directive*" OR "living will*" OR "ACP" OR "end of life care" OR "end of life plan*" OR "end of life healthcare" OR "end of life health care" OR "proactive health care" OR "proactive healthcare" OR "proactive care" OR "goals of care" ) OR AB ( "advance care plan*" OR "advance care directive*" OR "advance healthcare plan*" OR "advance health care plan*" OR "advance healthcare directive*" OR "advance health care directive*" OR "advance directive*" OR "living will*" OR "ACP" OR "end of life care" OR "end of life plan*" OR "end of life healthcare" OR "end of life health care" OR "proactive health care" OR "proactive healthcare" OR "proactive care" OR "goals of care" ) ) | Pub. date from 2010 – 2022 | 322 |

| **Database: CINAHL** | **Filters** | **Results** |
| --- | --- | --- |
| ( TI ( "end of life communicat*" OR "end of life convers*" OR "end of life discuss*" OR "end of life decision-mak*" OR "end of life talk*" OR “choices related to end of life” OR “choices related to death” OR “choices related to dying” OR “choices about end of life” OR “choices about dying” OR “choices about death” OR “communication about end of life” OR “communication about death” “communication about dying” OR “death and dying communication” OR “communication strategies about end of life” OR “communication strategies about death” OR “communication strategies about dying” ) OR AB ( "end of life communicat*" OR "end of life convers*" OR "end of life discuss*" OR "end of life decision-mak*" OR "end of life talk*" OR “choices related to end of life” OR “choices related to death” OR “choices related to dying” OR “choices about end of life” OR “choices about dying” OR “choices about death” OR “communication about end of life” OR “communication about death” “communication about dying” OR “death and dying communication” OR “communication strategies about end of life” OR “communication strategies about death” OR “communication strategies about dying”) ) **AND** ( TI ( "advance care plan*" OR "advance care directive*" OR "advance healthcare plan*" OR "advance health care plan*" OR "advance healthcare directive*" OR "advance health care directive*" OR "advance directive*" OR "living will*" OR "ACP" OR "end of life care" OR "end of life plan*" OR "end of life healthcare" OR "end of life health care" OR "proactive health care" OR "proactive healthcare" OR "proactive care" OR "goals of care" ) OR AB ( "advance care plan*" OR "advance care directive*" OR "advance healthcare plan*" OR "advance health care plan*" OR "advance healthcare directive*" OR "advance health care directive*" OR "advance directive*" OR "living will*" OR "ACP" OR "end of life care" OR "end of life plan*" OR "end of life healthcare" OR "end of life health care" OR "proactive health care" OR "proactive healthcare" OR "proactive care" OR "goals of care" ) ) | Pub. date from 2010 – 2022 | 571 |
