# Supplementary material for: Fundamentals of end-of-life communication as part of advance care planning from the perspective of nursing staff, older people, and family caregivers: a scoping review
Source: BMC Nurs. 2023 Oct 6;22:363. doi: 10.1186/s12912-023-01523-2 (PMC10559445; doi:10.1186/s12912-023-01523-2)
Supplement: Supplementary file 3 — Supplementary Material 3: Table C. Included study results and contribution per theme [file 12912_2023_1523_MOESM3_ESM.docx]

**Supplementary table C - “Included study results and contribution per theme”**

| Author (year) | Relevant primary results | Contribution to identified themes | | | | | | | | | | |
| --- | --- | --- | --- | --- | --- | --- | --- | --- | --- | --- | --- | --- |
|  | | *Person-centered approach* | *Preparing for EOL communication* | | | | *Carrying out EOL communication* | | *Professional attitude and required skills* | | | |
|  |  |  | Building a relationship with the conversation partner | Assessing the readiness of the older person | Timing and methods to start EOL communication | Practical requirements when preparing the EOL conversation | EOL communication based on information needs | Attention to the family relationship | Professional attitude | Improving EOL communication skills | Listening and non-verbal observation skills | Verbal communication skills |
| Almack et al. (2012) | *Factors identified by healthcare professionals:*  Factors that influence if EOL conversations are initiated:   - The need for training and developing experience in advanced communication skills - Judgement calls on the older person’s level of awareness or denial - Unwillingness of relatives to have these conversations - Uncertainty of trajectory with long term conditions   Factors that influence when EOL conversations take place:   - Older people initiate or ask for information - Judgement on timing - Once preparatory work is carried out (after getting to know the person, planning what to say) - Because of pressure to follow policy guidelines and find out older people’s preferences   Factors that influence how EOL conversations take place:   - Taking a ‘drip drip’ approach (a process in which something happens very slowly) - Use of trigger questions - Different choice of language | X | X | X | X |  | X |  |  | X | X | X |
| Groebe et al. (2019) | *Core subjects:*  EOL communication:   - Shared experience across settings - Experiences of a home hospice service - Experiences of a palliative care unit - Experiences of nursing homes - Barriers and need for support in communication   Topics in conversations (older people):   - Care planning and organizational matters - Life disclosure (the act of making something known) & life review - Spiritual and existential issues - Aging/old age (e.g., accepting closeness to death) - Bodily sensations as indication for attitudes   Topics in conversations (family caregivers):   - Care planning and organizational matters - Spiritual and existential issues - Questions about the dying process   Requirements and conditions of the daily care practice:   - Timing (When?) - Ways of having a conversation (How?) - Conversational partner (Who?)   Usefulness of tools: pro and contra:   - Contra perspective: counterintuitive to professional attitude - Contra perspective: no direct questions - Pro perspective: facilitate decision-making - Pro perspective: expressing thoughts - Pro perspective: mutual understanding to prevent conflicts | X | X | X | X | X | X | X | X | X | X | X |
| Hjorth et al. (2018) | The older person’s primary need facing EOL communication was “the comforting safety”, implying support, information, and transparency, with four core subjects:   - Provide good team-players (skilled communicators knowledgeable about treatment and the last phase of life) - Offer conversations with basic information - Preferred dialogues at the turning point in disease trajectory - Balance transparency | X | X | X | X |  | X | X |  |  |  |  |
| Isaacson et al. (2018) | EOL communication is orchestrated with the skilful interplay of the following communication patterns:   - Establishing context - Acknowledging through attentive listening - Making it safe for them to die - Planning goals of care - Being honest   These communication patterns are the nurse’s mechanism for attending to the completion of a life and are the overarching pattern - the closing composition. | X | X | X | X | X | X | X | X | X | X | X |
| Kerr et al. (2019) | *Core subjects:*  Feeling unskilled to have difficult conversations with older people who have life limiting illness:   - Absence of a communication toolkit and framework inhibits capacity to respond to difficult questions and gather information - Dealing with heightened emotions - Interactions are difficult when there is denial   Interacting with family caregivers adds complexity to care of older people who have life limiting illness:   - Unrealistic and mismatched expectations - Balancing relational dynamics with the older person’s values and needs   Organisational factors impede nurses' capacity to have meaningful conversations with older people and their family caregivers:   - Inadequate degree of information - Lack of time | X | X | X | X | X | X | X | X | X | X | X |
| Kimura et al. (2020) | EOL communication needs prioritized by nurses:   - Medical specialists who can provide mental and emotional support for older people and their family caregivers after EOL discussions. - Sufficient time is secured to have a talk. - Places that are suitable to have a talk in private with older people and their family caregivers. - Smooth cooperation with near-by palliative care wards and home-care services. - Opportunities from the early stage of treatment for physicians and older people to talk about the prospect of cancer treatment. - For the early stage of treatments, the palliative care team, psychiatrists, and psychologists can engage in the older person’s care on a continuing basis, as needed.   Highest rated roles and responsibilities   - Providing mental and emotional support for older people and their family caregivers after EOL discussion. - Verifying that older people and their family caregivers understand treatments, disease conditions, and prognosis after EOL discussion. - Repeating and/or supplementing explanation on treatments, disease conditions, and prognosis in a manner that older people and their family caregivers can understand after the EOL discussion. - Informing perceived medical conditions, family relations, fears and hopes of older people and their family caregivers to other members of the healthcare team before EOL discussion. - Suggesting setting up a time for an EOL discussion with other professionals involved in the older person’s care. - Assisting older people and their family caregivers in coping with social hardships. | X |  |  |  | X |  | X |  |  |  |  |
| Reinke et al. (2010a) | Nurses perceived that older people and family caregivers wanted their hopes to be supported and often provided this support independent of interactions with physicians. This can be described using the following core subjects:  Hope:   - Determine the individual’s meaning of hope - Focus on quality of life - Build trusting relationships with older people   Information:   - Assess what the older person knows about the disease/prognosis - ‘‘Follow the person’s lead’’ to determine their information needs   In contrast to the independent approach to addressing older people’s and family caregivers’ needs for hope, nurses emphasized their dependence on interactions with the older person’s physician when providing and supporting informational needs:   - Being informed about what the physician has said - Communicating information from older people back to physicians - Nurses continue discussions after the delivery of bad news | X | X | X |  | X | X |  |  |  | X |  |
| Reinke et al. (2010b) | The following items were endorsed as extremely important and also ranked as under-utilized:  Communication skills:   - Telling older people how their illness may affect their life - Giving enough information so that older people understand their illness and treatment - Talking with older people about what their dying may be like - Helping family caregivers understand what the dying process might be like - Talking with older people in an honest and straightforward way - Giving bad news in a sensitive way - Being willing to talk about dying - Being sensitive to when older people are ready to talk about death   Technical skills:   - Acknowledging and treating anxiety and depression - Knowledgeable about care older people need during the dying process - Helping older people and families understand how to provide symptom and pain control   Affective skills:   - Considering older people’s social situations when making treatment plans   Person-centered values:   - Not blaming or judgmental about lifestyles - Being comfortable with people who are dying   Person-centered care systems   - Avoiding keeping older people waiting without explanation - Minimizing interruptions and focusing on the older person during care - Making older people feel confident that they will not be abandoned prior to death - Helping older people and families get consistent information from healthcare team - Taking as much time as needed with older people | X |  | X |  | X | X | X | X | X | X |  |
| You et al. (2014) | Older people and family caregivers reported the same 5 guideline-recommended elements as most important:   - Ask the older person about preferences for care in event of life-threatening illness - Inquire about the older person’s values - Discuss prognosis - Give an opportunity for the older person to express fears or concerns - Ask the older person if they have additional questions about goals of care |  |  |  |  |  | X |  |  |  |  |  |
